# Thermal processing of food reduces gut microbiota diversity of the host and triggers adaptation of the microbiota: evidence from two vertebrates

**DOI:** 10.1186/s40168-018-0471-y

**Published:** 2018-05-31

**Authors:** Zhimin Zhang, Dapeng Li

**Affiliations:** 1 Department of Fishery Resources and Environment, College of Fisheries, Huazhong Agricultural University, Wuhan, People’s Republic of China; 2 Hubei Provincial Engineering Laboratory for Pond Aquaculture, Wuhan, People’s Republic of China

**Keywords:** Thermal treatment, Food, Vertebrate, Host selection, Gut microbiota

## Abstract

**Background:**

Adoption of thermal processing of the diet drives human evolution and gut microbiota diversity changes in a dietary habit-dependent manner. However, whether thermal processing of food triggers gut microbial variation remains unknown. Herein, we compared the microbiota of non-thermally processed and thermally processed food (NF and TF) and investigated gut microbiota associated with NF and TF in catfish *Silurus meridionalis* and C57BL/6 mice to assess effects of thermal processing of food on gut microbiota and to further identify the differences in host responses.

**Results:**

We found no differences in overall microbial composition and structure in the pairwise NF and TF, but identified differential microbial communities between food and gut. Both fish and mice fed TF had significantly lower gut microbial diversity than those fed NF. Moreover, thermal processing of food triggered the changes in their microbial communities. Comparative host studies further indicated host species determined gut microbial assemblies, even if fed with the same food. *Fusobacteria* was the most abundant phylum in the fish, and *Bacteroidetes* and *Firmicutes* dominated in the mice. Besides the consistent reduction of *Bacteroidetes* and the balanced *Protebacteria*, the response of other dominated gut microbiota in the fish and mice to TF was taxonomically opposite at the phylum level, and those further found at the genus level.

**Conclusions:**

Our results reveal that thermal processing of food strongly contributes to the reduction of gut microbial diversity and differentially drives microbial alterations in a host-dependent manner, suggesting specific adaptations of host-gut microbiota in vertebrates responding to thermal processing of food. These findings open a window of opportunity to understand the decline in gut microbial diversity and the community variation in human evolution and provide new insights into the host-specific microbial assemblages associated with the use of processing techniques in food preparation in humans and domesticated animals.

**Electronic supplementary material:**

The online version of this article (10.1186/s40168-018-0471-y) contains supplementary material, which is available to authorized users.

## Background

The emergence of metazoans has undoubtedly involved mutualistic relationships with diverse microorganisms that have presumably been a vital part of the evolution of vertebrates [1]. Indeed, co-evolution between humans and the microbiota in the gastrointestinal tract is a much discussed topic [2]. Diet is central to the evolution of modern humans [3] and other vertebrates, such as horses [4], and equally important in the evolution of their microbial communities [5]. Comparative studies of microbial communities are revealing factors that affect microbial diversity such as host genotype [6] and a range of environmental factors, particularly diet [7, 8].

The type and quantity of food consumed by modern humans are changing rapidly. The consumption of thermally processed (e.g., cooked) or sanitized foods is increasing due to their more effective digestion and prevention of infectious diseases. This largely contrasts with our closest primate relatives who continue to consume raw foods, unavoidably, where a large number of microorganisms colonize. Dietary shifts in humans over time have presumably occurred in three stages: from an increase in the sharing of plant roots, bulbs, and tubers in early *Homo* species [9] to an increased meat intake in *Homo sapiens* during the Pleistocene and to the adoption of agriculture practices and domestication of animals almost 10,000 years ago.

Dietary shifts result in specific changes in gut microbiota that can distinguish human populations based on their subsistence strategies (i.e., histories and lifestyles) [7]. Studies focusing on both urban-industrialized societies and traditional peoples with distinctly different dietary compositions indicate significant divergences in gut microbiota [7]. Recent work by Obregon-Tito et al. [10] explored the association between lifestyle and gut microbiota in hunter-gatherers and traditional agricultural communities in Peru and an urban-industrialized community in the US and showed that some microbes have been lost in urban-industrialized societies. Moreover, a progressive loss of gut microbial diversity has been demonstrated from the adoption of a low microbial-accessible carbohydrate diet over generations in mice [8].

Modern microbiota deviates substantially from our ancestors, and the diversity has decreased over time [11, 12]. In general, differences in gut microbiota between humans and other omnivorous primates are attributed to dramatic lifestyle changes such as the transition from raw to cooked food, farming, and industrialization. Thus, it is possible that microbial diversity and composition were altered at various key stages of human evolution [13]. There is a long evolutionary history of thermal processing in the diet throughout human evolution, from the first use of fire by our early ancestors to utilization of multiple cooking technologies in modern societies. Thermal processing is a socially unique human practice and represents a great advancement in food utilization. To date, many domesticated animals are also gradually transitioning to thermally processed food prepared by humans in industry-oriented developing societies. While the diet is an important mediator of gut microbial diversity, the potential role of thermal processing associated with food preparation in shaping gut microbiota has not been explored, other than the effect of shifting the diet alone. Given the evidence of an important role of thermal processing in the diet over human evolution, we hypothesized that the adoption of thermal processing in the diet has been an integral factor tailoring specific microbial structure signatures in modern humans and domesticated animals. However, it is indistinguishable that whether host responses that were elicited by thermal processing of food were evolutionarily conserved in the last common ancestor of all the animals or independently shaped in each host. Recently, host phylogeny has been reported to influence microbial community structure [6, 14], reflecting the conserved convergence of gut microbiota within a host. Thus, we further predicted diverse adaptive trajectories of gut microbiota in distantly different phylogenetic hosts such as aquatic and terrestrial animals to thermally processed food.

Mammals and fish species with evolutionally unique phylogenies are the most representative animals in our biosphere, living in ecologically different habitats. Some studies on interactions of host-gut microbiota and microbial differences in humans and animal models are documented [6, 14], though the microbial variation associated with thermal processing of food remains largely unclear. It is necessary to expand studies to this aspect in order to fully disentangle the gut microbial variation of the host and how the microbiome has co-evolved with the host. We selected both mice and fish to discern the effect of thermal processing of food on host-gut microbiota. On the one hand, mice contain a plenty of homologous genes in humans, favoring the more closely evolutionary conservation, which can help in understanding gut microbial changes in humans over the evolution; On the other hand, we further explored whether host responses are differently derived in terrestrial mammals and aquatic animals by comparing gut microbiota of mice and fish.

In this study, we targeted male C57BL/6 mice and southern catfish (*Silurus meridionalis*) that are capable of feeding on both non-thermally and thermally processed food to investigate the impact of thermal processing of food on gut microbial assemblages and compare the host responses of gut microbiota to thermally processed food. We provide evidence that thermal processing of food markedly dictates microbial diversity and community structure and that both animal hosts respond in a remarkably divergent way to thermally processed food.

## Methods

### Animal intervention

Four-week-old juvenile southern catfish from a pair of parents in a local fish farm were transported to the College of Fisheries, Huazhong Agricultural University, Wuhan, China. The fish were reared in the tank with the same culture condition for environmental adaptation for 1 week. Male C57BL/6 mice at 4 weeks of age were obtained from the Hubei Research Center of Laboratory Animals, Wuhan, China. All mice were kept in a cage for 1 week of adaptation with free access to water in an air-conditioned laboratory under the controlled experimental temperature (26 ± 2 °C). Grass carp (*Ctenopharyngodon idella*) (big fish) fillets and stone moroko (*Pseudorasbora parva*) (small fish) were used as the experiment foods in this study. Each food was used in duplicate: one as NF and the other as TF. The heating-up procedure to prepare the TF in a method of steaming was set for 15 min (the highest temperature, 100 °C; lasting 2~3 min). Lastly, four food groups, non-thermally and thermally processed grass carp (NG and TG) and non-thermally and thermally processed stone moroko (NS and TS), were obtained. The catfish and mice were supplied with their original food (water earthworm *Limnodrilus hoffmeisteri* for fish; chow for mice) and then transferred to combinations of original food and experimental food prepared for better dietary adaptation during the period of acclimatization. Experimental food supplies were increased gradually with the decline in original food supplies until the original food was totally replaced by the experimental food at the start of the experiments. Comparable catfish were randomly divided into four groups (*n* = 2 tanks/group) with separate non-recirculating water supplies in each tank (water temperature, 24.2~28.6 °C) to avoid cross-contamination of microbiota; meanwhile, mice were divided into two groups (*n* = 2 cages/group). NG and TG were supplied to two groups of catfish (named as F_NG and F_TG) and two groups of mice (M_NG and M_TG). And two other groups of catfish were supplied with NS and TS (F_NS and F_TS), respectively. The uneaten food residues were removed from the tanks within 1 h after fish feeding and from the cages within 2~3 h for mice. Both animal species were fed three times daily (in the morning, dusk, and midnight). The fish and mice experiments lasted 8 and 9 weeks, respectively. The body weight of the fish and mice were measured at the end of the experiment (Additional file 1: Table S1).

### Sample collection

In this study, the genetic backgrounds of southern catfish from the same broodstocks and the identical culture conditions to the largest degree reduce individual variations in gut microbial community. Thus, we did not collect gut samples prior to experiments. By contrast, it is not sure whether mice had the same parents despite the same age. In order to assess if early microbial variations among individuals confuse the late resultant differences, four fecal samples were randomly collected from mice before experimental food intervention (named as M_BA).

Grass carp were purchased from a local market and stone moroko were obtained from Lake Liangzi as the experimental foods. Grass carp were dissected with the viscera, and the head was removed. Paired NF and TF (NG vs TG; NS vs TS) samples were collected about every 2 weeks during the experimental periods for analyses of food microbiota and nutritional characteristics including proximate composition, fatty acids, and amino acids. The NS group of four samples was contaminated, so the samples were not used in subsequent sequencing. The catfish were fed two food sources with different treatments (NG vs TG; NS vs TS) for assessing the effect of food type, food treatment, and their interactions on microbial assemblies. At the end of the experiments, four catfish from two tanks in each group (*n* = 2 fish/tank) were killed with an overdose of anesthetic MS-222. The posterior intestine (approximately half of the intestinal tract) was removed after dissection using a sterile scalpel and forceps. Similarly, four mice from two cages in each group (*n* = 2 mice/cage) were separately caged into four sterile cages for fecal sampling. In total, four samples from each grouped fish and mice were obtained for microbial analysis. All samples were separately placed in sterile 1.5 ml tubes and stored at − 80 °C until analysis.

### DNA extraction of microbial samples

DNA was extracted from all samples using a QIAamp DNA Stool Mini Kit (Qiagen, Hilden, NRW, Germany) following the manufacturer’s instructions with modifications. In brief, 1 ml of lysis buffer was added to ~ 100 mg fecal samples or ~ 200 mg gut and food samples and then vortexed horizontally until homogeneity was achieved. The samples were incubated at 95 °C for 5 min and centrifuged for 2 min at full speed. The resulting DNA pellets were dissolved in 120 μl TAE buffer. The final DNA concentration was determined using NanoDrop ND-2000 (ThermoFisher Scientific, Hudson, NH, USA), and DNA quality was evaluated by agarose gel electrophoresis.

### Amplification and sequencing of bacterial 16S rRNA genes

The V4–V5 hypervariable regions of the 16S rRNA genes were amplified with primers 515-Forward (5′-GTGCCAGCMGCCGCGGTAA-3′) and 907-Reverse (5′-CCGTCAATTCCTTTGAGTTT-3′). A specific primer with unique barcodes was used for identifications of different samples. PCR was performed in triplicate with 15 μl of Phusion High-Fidelity PCR Master Mix (NEB, Ipswich, MA, USA), 0.2 μM of forward and reverse primers, and 10 ng of template DNA per 30 μl reaction. Thermal cycling consisted of an initial denaturation step at 98 °C for 1 min, followed by 30 cycles of denaturation at 98 °C for 10 s, annealing at 50 °C for 30 s, and elongation at 72 °C for 30 s, with a final elongation at 72 °C for 5 min. Identical PCR products were combined in equal amounts, and the mixtures were purified with GeneJET Gel Extraction Kit (ThermoFisher Scientific, Hudson, NH, USA). Sequencing libraries were constructed using an NEB Next UltraTM DNA Library Prep Kit (NEB, Ipswich, MA, USA) for Illumina (San Diego, CA, USA) following the manufacturer’s recommendations, and index codes were added. Library quality was assessed using a Qubit 2.0 Fluorometer (ThermoFisher Scientific, Hudson, NH, USA) and Agilent Bioanalyzer 2100 system (Agilent, Santa Clara, CA, USA). The resulting amplicons were sequenced on the Illumina HiSeq 2500 platform.

### Sequence processing, taxonomy assignments and community structure analyses

Raw sequence data were processed using QIIME Pipeline-Version 1.7.0 [15]. All sequences were trimmed and assigned to each sample based on their barcodes (barcode mismatches = 0). Overlapping paired-end reads were merged using FLASH-1.2.8 software [16]. Merged sequences (read length > 300 bp, without ambiguous base “N,” and average base quality score > 30) were used for further analysis. All sequence reads were sorted based on their unique barcodes. Chimeric sequences were removed using the UCHIME algorithm [17]. Sequences were subsampled to the same sequence depth using daisychopper.pl [18] for downstream analysis. Sequences were clustered into operational taxonomic units (OTUs) with CD-HIT algorithm using a 97% identity cutoff, and singletons were removed. Phylogenetic affiliation sequences were analyzed by the Ribosomal Database Project classifier [19]. Assessments of within-community diversity (alpha diversity) and between-community diversity (beta diversity) were implemented in QIIME with in-house Perl scripts. Alpha diversity was estimated using four different metrics including Shannon and Simpson indices for biodiversity and observed species and Chao1 for microbial species richness. In addition, we compared microbial compositions of top 50 OTUs between the experimental food and gut using UPGMA method based on Bray-Curtis distances together with a heat map of abundance data and further visualized gut samples for beta-diversity analysis using Principal Coordinate Analysis (PCoA) based on UniFrac matrices. To further explore key phylotypes that may contribute to the observed differences in microbial communities, linear discriminant analysis (LDA) effect size (LEfSe) algorithm was performed (http://huttenhower.sph.harvard.edu/galaxy) combining Kruskal-Wallis test or Wilcoxon rank-sum test with LDA scores to estimate the effect size of differentially abundant features with biological consistency and statistical significance (herein, *α* value for the statistical test was set at 0.05 and threshold on the LDA score for discriminative features was more than 3.0).

### Statistical analysis

Before data analysis, Shapiro-Wilks test was used to verify homogeneity of variance. Student’s *t* test was used to detect differences in proximate composition and alpha diversity of paired food groups when data met the homogeneity of variance; otherwise, Welch’s *t* test or unequal variance *t* test was used. Differences in profiles of overall fatty acids and amino acids of the two foods with different treatments were statistically analyzed using two-way permutational multivariate analysis of variance (PERMANOVA). To evaluate sample dispersion within groups, we calculated inter-sample dissimilarity based on weighted UniFrac distance and tested the significance within groups using one-way ANOVA with Tukey’s HSD post hoc test. Differences in beta-diversity of gut microbiota of mice before and after food intervention were statistically assessed using one-way PERMANOVA. Further, host species and food treatment effects on gut microbiota were evaluated using two-way PERMANOVA. Herein, we contrasted “mice vs fish” and “non-thermally processed food vs thermally processed food” and tested for their interactive effects. Similarly, to detect whether there were food type and thermal treatment effects on gut microbiota in fish, we also used the method where “grass carp fillets and stone moroko” and “non-thermally processed food vs thermally processed food” were contrasted. All univariate testing and all multivariate testing with 9999 permutations were performed in SPSS Statistics 20 and Past 3.0, respectively. A *p* value < 0.05 was considered statistically significant.

## Results

### Biochemical compositions of experimental food

The proximate composition of the food is shown (Additional file 1: Table S2). Thermal processing had no effects on fat and ash contents in the two foods (*p >* 0.05 for both). The thermal processing decreased water content of grass carp fillets and stone moroko (*p <* 0.001 for both). It caused a slight decrease of protein content for grass carp fillets (*p <* 0.01), but not for stone moroko (*p >* 0.05, Additional file 1: Table S2). Regardless of thermal processing, the protein content in grass carp fillets was higher compared to stone moroko (on average, 15.44 vs 13.01%, *p <* 0.001), but the ash content was lower (1.26 vs 3.14%, *p <* 0.001). The two experimental foods contained low and similar levels of fat contents (on average, 2.22% for grass carp fillets and 2.27% for stone moroko, *p* > 0.05) and had no differences in water contents (*p* > 0.05).

We further analyzed the profiles of fatty acids and amino acids of the experimental foods. Principle Component Analysis (PCA) based on fatty acid profiles showed that thermal treatment did not result in overall fatty acid changes (two-way PERMANOVA, *p >* 0.05, Additional file 1: Figure S1a) and that food samples clustered according to food type (*p <* 0.001). Based on amino acid profiles, PCA also showed sample separations of grass carp fillets and stone moroko (two-way PERMANOVA, *p <* 0.05, Additional file 1: Fig. S1b) were not dependent on food treatment (*p >* 0.05). These results indicate that food type predominantly affects the profiles of fatty acids and amino acids rather than thermal treatment in this study.

### Overview of high-throughput sequencing data

We characterized 28 gut microbiota samples from four groups of catfish (F_NG, F_TG, F_NS, and F_TS) and three groups of mice (M_BA, M_NG, and M_TG), and 12 food microbiota samples from three food groups (NG, TG and TS). In total, 2,549,512 raw sequences were obtained. After performing quality trimming and chimera checking, we obtained 2,028,760 high-quality processed sequences with a mean length of 371 bp, accounting for 80% of all valid sequences, with an average of 50,719 sequences (ranging from 29,083 to 63,992) per sample. To minimize bias due to sequencing depth, we normalized the sequence number against the sample with the lowest sequence number obtained by random subsampling. High coverage values (average = 99%) were obtained for sequences in all samples, indicating that the sequencing depth was sufficient.

### Thermal processing does not significantly affect food microbiota

**Fig. 1:**
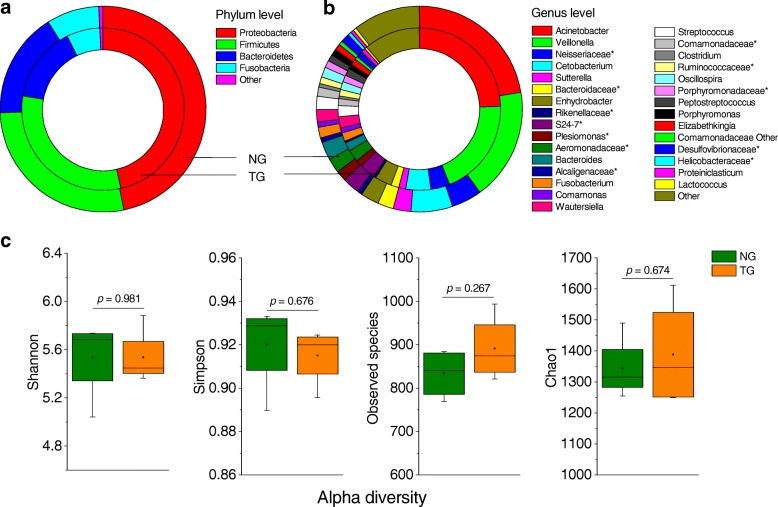
Microbial relative abundance and alpha diversity in the food. Microbiota in food, grass carp fillets, fed both the catfish, and mice was assessed. Donut charts of the relative abundance at the **a** phylum and **b** genus levels. Outer and inner donuts represent the relative abundance in non-thermally processed and thermally processed grass carp (NG and TG) food, respectively. **c** Multiple indices for alpha diversity estimation. The symbol “*” denotes unclassified OTUs at a taxonomic higher or lower level

Microbial taxonomic compositions of three groups of experimental foods are showed in Additional file 1: Figure S2. The results identified that dominant phyla were *Proteobacteria*, *Firmicutes*, *Bacteroidetes*, and *Fusobacteria*, together accounting for an average of 99.3, 99.4, and 98.8% of all classifiable sequences in NG, TG, and TS, respectively. TS group had higher abundance of *Proteobacteria* and lower abundance of *Firmicutes* (Additional file 1: Figure S2a) compared to NG and TG groups. There were no significant differences in the abundance of each phylum between NG and TG groups (Fig. 1a, Additional file 1: Figure S2a). At the genus level, *Acinetobacter* and *Veillonella*, followed by *Cetobacterium* and unclassified *Neisseriaceae*, dominated in microbial communities of NG and TG groups*,* which consisted of approximately half of all the sequences (Fig. 1b, Additional file 1: Figure S2b). Despite these taxa being detected in TS, the compositional abundances differed from those in NG and TG groups. *Halomonas* was the most abundant genus in TS group (13.5%), but it was significantly lower in NG (0.5%) and TG (0.5%) groups (Additional file 1: Figure S2b). As shown in Additional file 1: Figure S2c, TS group separated from NG and TG groups (weighted UniFrac, one-way PERMANOVA, *p <* 0.001), but the two groups were not (*p >* 0.05). Similarly, for alpha diversity, there were no significant differences between NG and TG groups (*p >* 0.05 for all alpha diversity metrics, Fig. 1c).

### Thermal processing of food decreases gut microbial diversity

**Fig. 2:**
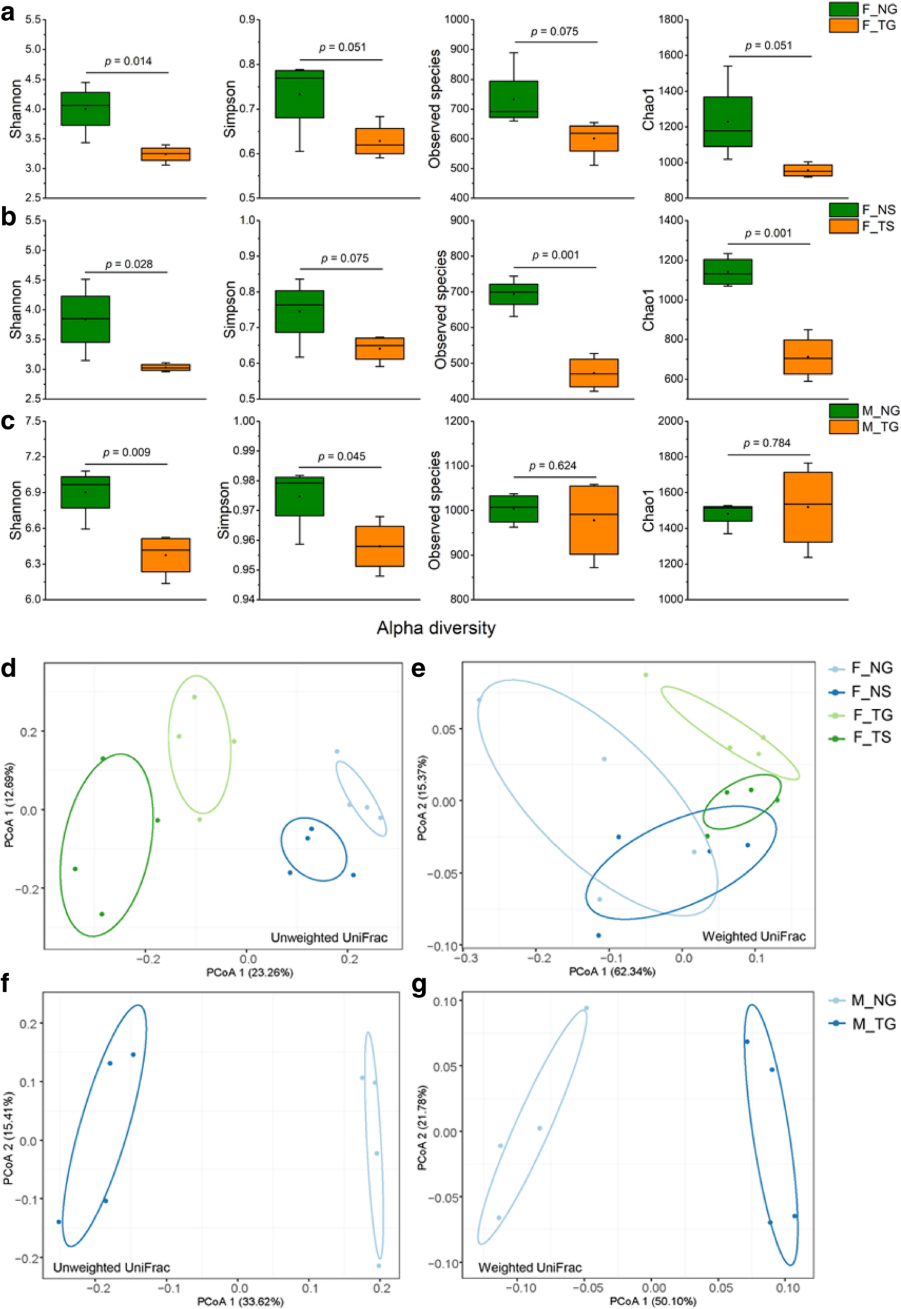
Thermal processing of food affects gut microbial community of the catfish and mice. Thermal processing of food decreases alpha diversity of the microbial community of catfish fed **a** grass carp fillets and **b** small stone moroko, and of mice fed **c** grass carp fillets. Principal coordinate analysis based on unweighted UniFrac distance for **d** catfish and **e** mice, and weighted UniFrac distance for **f** catfish and **g** mice shows that thermal processing of food induces significant changes in the gut microbial community of the catfish and mice

TG led to significantly lower Shannon index diversity in the catfish gut (*p <* 0.05) and had near-significant effects on reduced species evenness (*p* = 0.051 for Simpson index) and species richness (*p* = 0.075 for observed species and *p* = 0.051 for Chao1) compared with NG (Fig. 2a). In line with these results, overall lower alpha diversity were observed in F_TS than F_NS (*p* < 0.05 for Shannon index, *p* = 0.075 for Simpson index, *p* < 0.01 for observed species and Chao1; Fig. 2b). Similarly, alpha diversity measurements revealed lower values of Shannon and Simpson indices in M_TG compared to M_NG (*p <* 0.05 for both; Fig. 2c). No shifts in microbial species richness were indicated by observed species and Chao1 (*p >* 0.05 for both; Fig. 2c). Overall, these results reveal that thermal processing of food results in general decreases in gut microbial diversity.

### Thermal processing of food alters gut microbial community

**Fig. 3:**
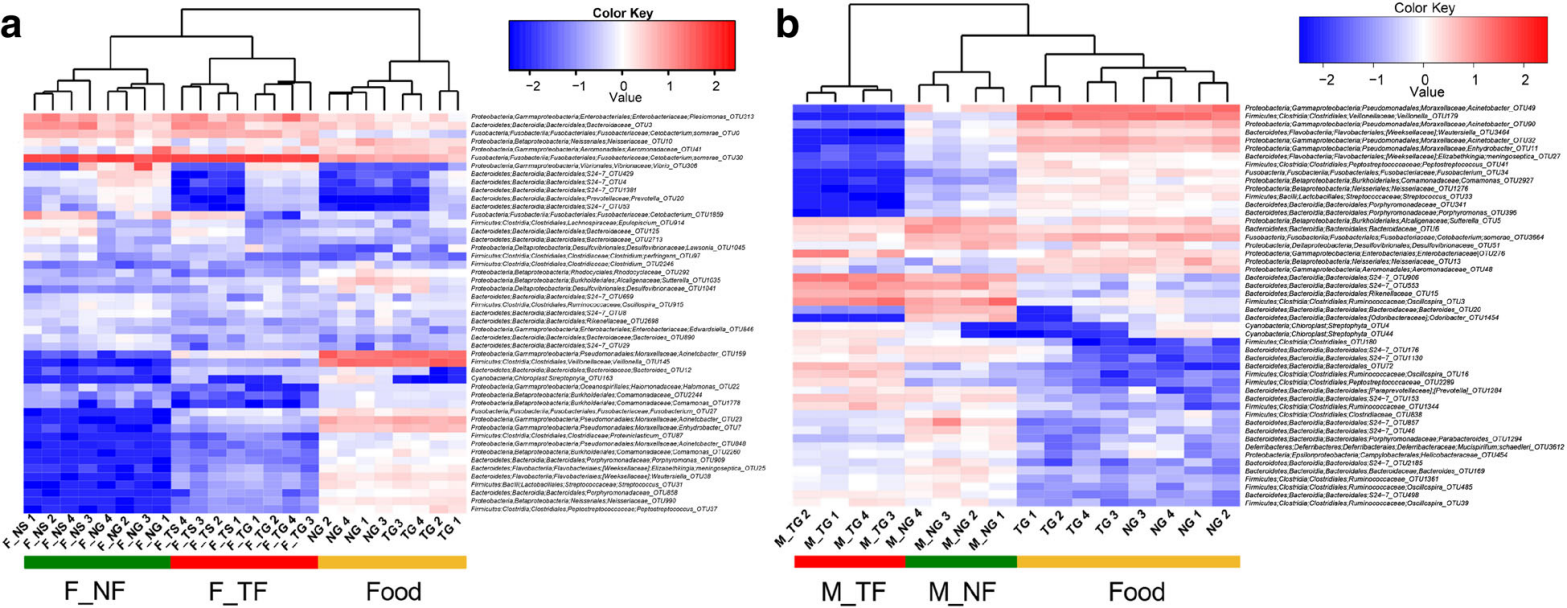
Hierarchical clustering dendrogram of microbiota in the gut and food. Dendrograms of gut microbiota in **a** catfish and **b** mice coupled with microbiota of food simultaneously supplied to the catfish and mice for the top 50 genera, based on Bray-Curtis distance metrics. The heat map depicts the relative abundance of each genus in each sample. The color scale for the heat map is displayed in the upper right corner of the figure

Gut microbial communities differed in mice before and after food intervention (weighted UniFrac, one-way PERMANOVA, *p <* 0.001; Additional file 1: Figure S3a); however, intra-group microbial dissimilarities did not change (one-way ANOVA, *p >* 0.05; Additional file 1: Figure S3b). Using catfish and mice fed NG and TG to assess sources of gut microbial differences, the results showed the overall microbial community structure was strongly associated with effects of host species (weighted UniFrac, two-way PERMANOVA, *p <* 0.001), food treatment (*p <* 0.01), and their interactions (*p <* 0.05) (Table 1). The main effects and the interactive effects were also found on microbial members (Additional file 1: Table S3). Further, using catfish fed grass carp fillets (NG and TG) and stone moroko (NS and TS) to assess effects of food treatment, food type and their interactions on fish gut microbiota, we found that food treatment (weighted UniFrac, two-way PERMANOVA, *p <* 0.001) and food type (*p <* 0.01) significantly contributed to changes in microbial structure, yet no significant effects were detected for their interactions (*p >* 0.05) (Table 2). In addition to food treatment and food type, their interactions disclosed significant effects on fish gut microbial members (Additional file 1: Table S4). PCoA was used to visualize an overview of gut microbial communities in catfish or mice fed with the paired food at the OTU level. Separation was clear in both microbial members (unweighted UniFrac, Fig. 2d), and microbial structure (weighted UniFrac, Fig. 2e) for catfish according to their food treatments. This pattern also occurred in mice (unweighted UniFrac, Fig. 2f; weighted UniFrac, Fig. 2g). Clustering analysis based on Bray-Curtis metrics of the top 50 genera further confirmed the distinctness of gut microbial communities: both catfish and mice samples clustered together 100% of the time according to their treatment group (Fig. 3). In catfish, a distinct sub-cluster nested within different food sources with the same food treatment was observed (Fig. 3a). In addition, food had an approximate clustering that was clearly separated from that of catfish (Bray-Curtis, one-way PERMANOVA, *p <* 0.001, Fig. 3a) and mice (*p <* 0.001, Fig. 3b). These results suggest that, in addition to host species and food itself, thermal processing of food also shapes gut microbial communities in vertebrates.

**Table 1:** Two-way PERMANOVA based on weighted UniFrac distance testing whether gut microbial communities have differences between mice and catfish fed non-thermal and thermal processing grass carp fillets

Source	d.f.	SS	MS	Pseudo-*F*	*p*
Host	1	0.6571	0.6571	211.71	0.0001
Treatment	1	0.0272	0.0272	8.7553	0.0087
Interaction	1	0.0289	0.0289	9.3063	0.0105
Residual	12	0.0372	0.0031		
Total	15	0.7504

**Table 2:** Two-way PERMANOVA based on weighted UniFrac distance testing whether gut microbial communities have differences in catfish fed grass carp fillets and stone moroko with non-thermal and thermal processing

Source	d.f.	SS	MS	Pseudo-*F*	*p*
Treatment	1	0.1285	0.1285	7.3559	0.0002
Diet	1	0.0816	0.0816	4.6703	0.006
Interaction	1	0.0267	0.0267	1.5256	0.1943
Residual	12	0.2097	0.0175		
Total	15	0.4464

### Gut microbiota differentially responds to thermal processing of food

**Fig. 4:**
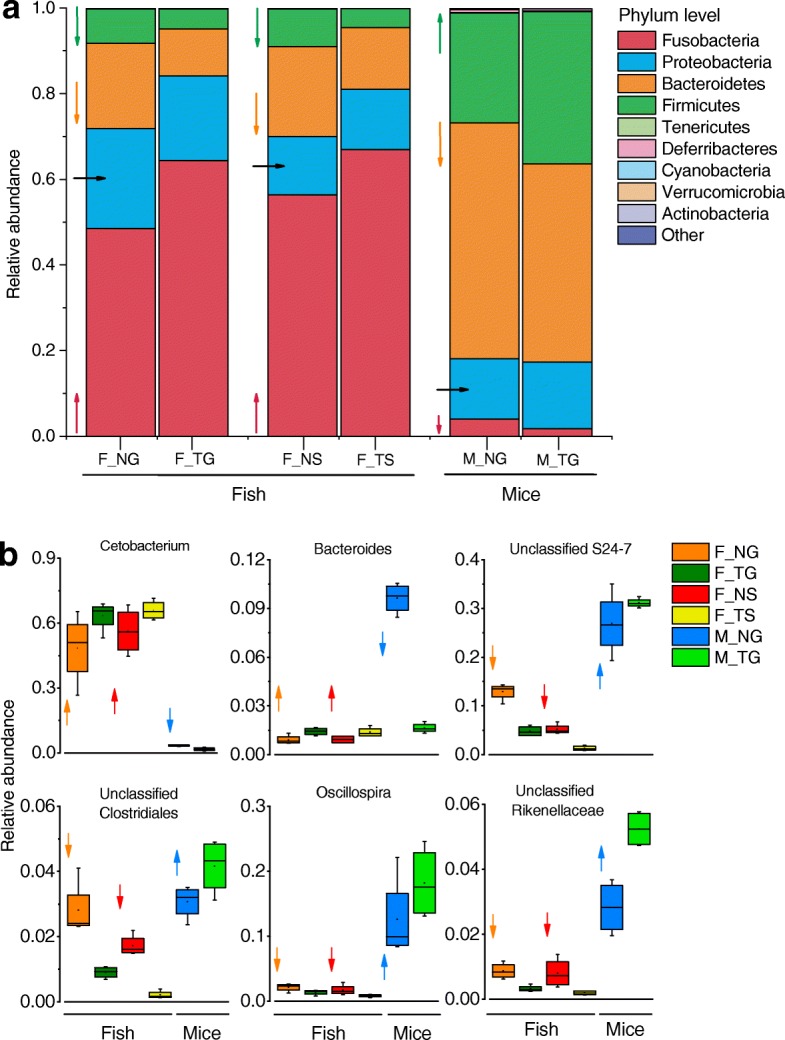
Taxonomic compositions and changes of gut microbiota in the catfish and mice. Relative abundance and the change trend caused by thermally processed food of dominant gut microbiota at the phylum level (**a**) and genus level (**b**). The arrow indicates the change trend of the gut microbiota in thermally processed food-fed catfish or mice compared to that in the corresponding non-thermally processed-fed counterparts. ↑ increase, → stable, ↓ decrease

Gut microbial taxa at the phylum level were dominated by *Fusobacteria*, *Proteobacteria*, *Bacteroidetes*, and *Firmicutes* in catfish and mice (Fig. 4a, Additional file 1: Figure S3c). Microbial samples from mice before food intervention were sequenced and were compared to those after the intervention (Additional file 1: Figure S3c). The most abundant taxon in all mice was *Bacteroidetes* (56.2%), followed by *Firmicutes* (27.9%). The abundance of *Bacteroidetes* was higher in M_BA (67.3%) compared to M_NG (55%) and M_TG (46.3%), whereas there was a lower level of *Proteobacteria* in M_BA (6.8%) than M_NG (14.1%) and M_TG (15.5%). However, thermal processing of food increased the abundance of *Firmicutes* in mice (M_NG = 25.6% and M_TG = 35.6%). No differences in *Proteobacteria* were observed between M_NG and M_TG, similar to the results obtained in two different paired groups of catfish (Fig. 4a). *Fusobacteria* dominated in the gut of catfish, with a decreased abundance in F_NG compared to F_TG (48.5 vs 64.4%, Fig. 4a). The reduction was also observed in F_NS compared to F_TS (56.4 vs 66.9%). By contrast, F_NG and F_NS had higher abundant *Bacteroidetes* (20.0 vs 11.0% and 21.0 vs 14.4%) and *Firmicutes* (7.9 vs 4.7% and 8.8 vs 4.5%) than the corresponding groups. The *Firmicutes*-*Bacteroidetes* ratio increased significantly in M_TG than M_NG (*p <* 0.05, Additional file 1: Figure S4). The ratio was stable between F_NG and F_TG (*p *> 0.05) and decreased in F_TS compared to F_NS (*p <* 0.05). Figure 4b shows the predominant taxa in catfish and mice at the genus level. Thermal processing of food resulted in increased trends of the most abundant genera, such as *Cetobacterium* (F_NG = 48.5% and F_TG = 63.4%; F_NS = 56.3%, F_TS = 65.9%) in fish, and unclassified *S24-7* (M_NG = 26.9% and M_TG = 31.1%) and *Oscillospira* (M_NG = 12.6% and M_TG = 18.2%) in mice (Fig. 4b, Additional file 1: Figure S3d). These taxa disclosed the opposite changes in fish and mice. Similarly, the *Bacteroides* significantly increased in fish by thermal processing of food (F_NG = 0.9% and F_TG = 1.4%; F_NS = 0.9% and F_TS = 1.4%), whereas it decreased dramatically in mice (M_NG = 9.6% and M_TG =1.6%); meanwhile, this observation was suitable to unclassified *Rikenellaceae* in both the animals (Fig. 4b).

**Fig. 5:**
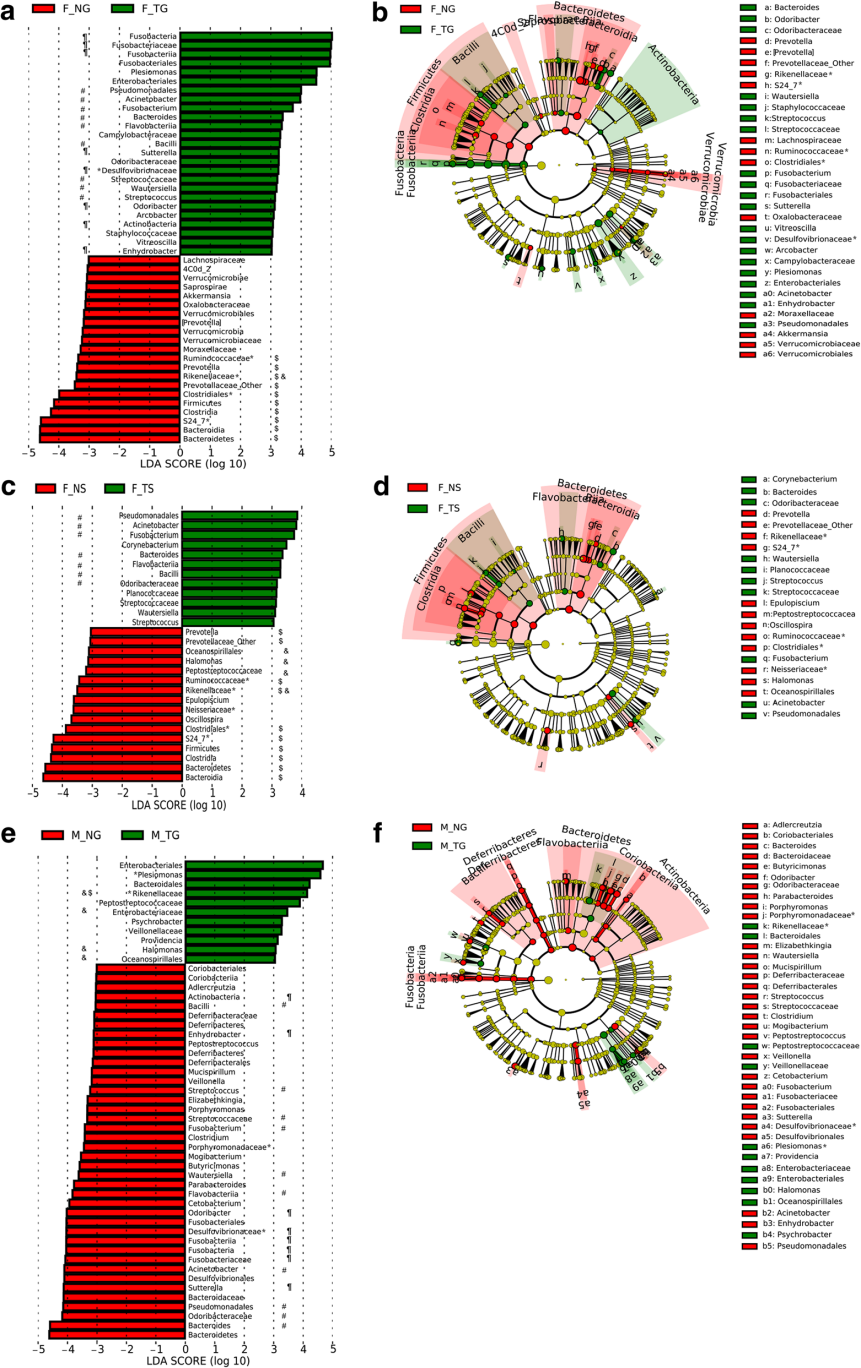
LEfSe analysis identifying taxonomic differences in the gut microbiota of the catfish and mice responding to thermally processed food. Key phylotypes of differently abundant taxa were identified using linear discriminant analysis (LDA) combined with effect size (LEfSe) algorithm. Histograms of LDA scores of 16S gene sequences in F_NG and F_TG (**a**), F_NS and F_TS (**c**), and M_NG and M_TG (**e**) are shown, with a cutoff value of LDA score (log_10_) above 3.0. **a**, **c*,*** and **e** F_NG, F_NS, and M_NG-enriched taxa are indicated with a negative LDA score (red), and taxa enriched in the F_TG, F_TS, and M_TG are characterized by a positive score (green). The symbols “# and ¶” denote enriched taxa in the F_TG and/or F_TS; however, those in the M_NG, where the symbol “#” simultaneously denotes the same enriched taxa in F_TG, F_TS, and M_NG; the symbols “& and $” denote enriched taxa in the F_NG and/or F_NS, but those in the M_TG, where the symbol “$,” denotes the same enriched taxa in the F_NG and F_NS. **b**, **e**, and **f** Cladograms are derived from LEfSe analysis of differential gut microbial taxa. The central point denotes the root of the tree of bacteria and expanded to each ring representing the next lower taxonomic level from phylum to genus. Each circle’s diameter represents the relative abundance of the taxon in gut microbial community. The symbol “*” denotes unclassified OTUs at a taxonomic higher or lower level

The strict version (all against all) of LEfSe was assigned to robustly identify abundant microbial taxa with a log LDA score above 3.0 that were statistically different between biological classes in this study. LEfSe analysis revealed 25 and 21 phylotypes in F_NG and F_TG (Fig. 5a), 12 and 16 phylotypes in F_NS and F_TS (Fig. 5c), and 11 and 40 phylotypes in M_NG and M_TG (Fig. 5e) for distinguishing taxonomic differences between the paired groups of catfish and mice. Of the phylotypes in fish, ten were simultaneously enriched in F_NG and F_NS; meanwhile, ten were overrepresented in F_TG and F_TS. As shown in the biologically clades (Fig. 5b, d, and f), taxonomic distributions further confirmed specific gut microbial taxa from phylum to genus associated with thermal processing of food, such as the overrepresented *Firmicutes* and *Fusobacteria* in TF-fed fish and the overrepresented *Bacteroidetes* in NF-fed fish (Fig. 5b, d). Moreover, many specific abundant taxa in NF-fed fish were overrepresented in TF-fed mice, and vice versa (Fig. 5a, c, and e), indicating opposite patterns of gut microbial enrichment between fish and mice responding to thermal processing of food. The differential shifts were also observed at the OTU level (Fig. 3). These results strongly suggest host-specific alterations of gut microbiota by thermal processing of food.

## Discussion

The factors mediating community assembly and structure are of a hotspot in microbial ecology in terrestrial and aquatic animals. The key roles of microbial communities are associated with the development and acclimation of host to environmental changes [1, 2]. Diet is known to drive host evolution and affect gut microbiota assemblages [3–5]. In this study, the results further exhibited that thermal processing of food effectively resulted in a reduced gut microbiota diversity of both fish and mice and affected their microbial communities, with extensively distinct responses of symbiotic gut microbiota between the hosts. The findings corroborate our hypothesis that thermal processing of food drives the assembly of complex gut microbiota in vertebrates associated with host-specific adaptive selection.

We constrained the most possible diet effects by providing mice and catfish hosts with the same food so that microbial changes observed could be correlated with the effect of the host. The gut microbiota of mice and catfish almost shared the same microbial divisions, although the dominating taxa within the divisions obviously differed. In mice, *Bacteroidetes* and *Firmicutes* were the two most important divisions, with the majority of microbial phylotypes; in contrast, gut microbial community of the catfish was composed mainly of *Fusobacterium* and *Proteobacteria*. These results are broadly similar to those of gut microbiota in murine and teleost fish [20]. The extensive divergences in gut microbiota between mice and fish strongly indicate that host species determines an essential role in microbial assemblages [6], consistent with observations from previous studies of different mammals [21] and other wild and domesticated animals [22, 23]. In contrast to host genetics as the endogenous determinant of gut microbiota, food is an important exogenous driving factor for microbial configurations. The meticulous comparison in the catfish fed two different food sources with thermal and non-thermal processing revealed differential gut microbiota within intra-species host, confirming the effect of food on gut microbial composition and structure. The alterations might be associated with the differences in macro- and micro-nutrients of food components. Notably, the significantly higher ash content in stone moroko food containing much fishbone is assuredly related to abundant of mineral elements, especially calcium, which can manipulate gut microbiota [24, 25].

Our study centered on whether the impact of the gut microbiota is facilitated by thermal processing of food. As expected, thermal processing significantly altered the communities concurrently in fish and mice. Unlike commercial foods consumed by humans and many domesticated animals that are usually less enriched in microbiota and even sterile due to heavily heated processing, it is easy to carry some microbiota from the surrounding environment when food is unprocessed or slightly processed. Thus, we intentionally aimed to detect the microbiota of food used in this study and then excluded the potential influences of microbial structure of food on the communities in the gut because of an unchanged alpha diversity and stable microbial community observed in the paired NF and TF. Unfortunately, the results seem to provide no conclusive evidence that the gut microbial alterations of the mice and fish are unrelated to the food microbiota. The reason for this is due to thermal processing might diminish or kill microbial activities that may alter the microbial interactions between the gut and food. However, the downfall is intrinsic to thermal processing of food, and it occurs in sequencing based on microbial ecology studies. In general, small proportions of gut microbiota were derived from the microbiota present in food and other surrounding environments, which has been documented in some studies focusing on different animals [26, 27]. This is also supported by dramatic differences in microbial abundances and compositions between the two animals’ gut and their foods in the present study. Thus, it could be inferred that the differences in gut microbiota of the host responding to the pairwise food are mainly driven by thermal processing of food rather than food microbiota.

Convergent shifts in gut microbial diversity were observed in two groups of TF-fed catfish. An increase of highly abundant microbiota in host, such as *Cetobacterium* in F_TG and F_TS, is likely responsible for a decrease of other taxa and eventually leads to a low microbial diversity. Supporting this, a similar phenomenon was found in M_TG. Likely, thermal processing of diet dysregulates the competitive mechanisms of the less abundant species downregulated by the diet, which then releases the dominant microbiota from competitive exclusion, enabling it to expand in abundance. The emerging picture appears to follow the community ecological theory, which predicts that highly abundant species monopolize most of the resources in the habitats, and over time accelerate the reductions and extinctions of rare species in communities with initial uneven abundance patterns compared with more even abundance patterns [28]. Dietary intervention can improve low gene richness of gut microbiota, which has the potential to benefit the host [29]. Conversely, it also plays negative roles in gut microbial assembly. The human microbial diversity is greatly depleted compared with our closest living ape relatives [11]. As proposed by Gillings et al. [13], a history of a series of ecological and evolutionary drivers strongly contributes to the declining microbial diversity over human evolution, and the use of fire in diet preparation is presumed as an initial factor lowering the diversity. Herein, we present the first evidence that thermal processing of food markedly decreases the microbial diversity in the gut of two vertebrates, providing new insights into the sharp decreases of the microbial diversity in humans.

As hypothesized, our results indicate that TF triggers diverse taxon-specific inter-species changes. Compared to NF-fed individuals, TF-fed counterparts maintained or reduced the ratios of *Firmicutes* and *Bacteroidetes* in catfish, while an elevated ratio occurred in TF-fed mice with more body weight (Additional file 1: Table S1). The resulting changes in mice match the previous results observed in obese individuals compared to their lean counterparts in human [30]. Such a trend is likely to generate correlations between diet-related gut microbiota and host fitness [31], but diverse patterns are driven by different populations responding to a Western diet [32]. As detected in more details at the genus level, in this study, we found opposite patterns of changes in many dominant taxa between fish and mice, which has not been previously reported in different hosts fed the same diet. A simple explanation is that the effects of thermal processing of food on gut microbiota rely upon host phylogeny and/or that differences in host-specific response to thermal processing of food shaping gut microbiota are likely to be genetically driven. This further highlights that host and thermal processing of food would interact for modulating gut microbiota. More intriguingly, this raises several questions: whether different host responses to TF apply to other populations and if different microbial changes correlate to microbial function redundancy for the host during the adaptation. More research is needed, not only on representative mammals such as humans and closely related mice family but also on commonly domesticated animals, such as poultry and salmons, to better understand the patterns present in this study.

Generally, gut microbial communities vary geographically with the host in large part because they are suited to local diet and lifestyle and the local adaptation could be enhanced by incorporation and acquisition of exogenous genes by gut microbiota [33]. Changes in early hominid diet came with the adoption of cooking, yet it is almost impractical to obtain direct evidence associated with cooking or processed foods shaping gut microbiota over human evolution because few fecal samples in early human history are available. Nevertheless, our results might support that divergences of gut microbiota correlate to the diversified dietary habits. For example, some people in a dietary culture show a preference for plant-based food, and some in other cultures consume meat-heavy food, even non-thermal processed such as sashimi and raw beef. When a diet consists largely of TF that can produce end products toxic to humans, such as acrylamide and ammonia, due to Maillard reaction during the heating, thermal processing may have shaped assemblages of human gut microbes for adapting to the gut ecosystem by introducing products of the Maillard reaction which can be further degraded by the resident microbiota. In in vitro gut model, Tuohy et al. (2006) found heated protein reduced the numbers of beneficial microbiota such as *Bifidobacteria* and *Lactobacilli* and increased the numbers of detrimental microbiota [34], supporting the effect of cooked food on gut microbiota. Particularly significant is the finding that human gut bacterial enzymes could degrade xenobiotics unique to TF [35]. Therefore, thermal processing that is incorporated into dietary transitions from a very early hominid diet, to a Neolithic diet, and to today’s typical of West diet rich in high protein and fat foods, such as red meat and baked potatoes and coffee, further promotes the assembly of the present-day human gut microbiota.

An alternative perspective to microbial alterations by TF is that an increased energy intake is triggered by an incorporation of animal products such as cooked meat into diets that appears to have accelerated the human evolution in terms of the morphological development, resulting in larger brain and body size, and smaller gut [36, 37]. Similarly, Carmody et al. (2011) found that thermal processing significantly increases energy intake and leads to larger individuals in mice [38]. These imply that TF modulates gut microbial communities to alter host energy intake and fat deposition [39]. The correlation between altered gut microbiota and diet [40] to large degree could be considered as a consequence of the interactions between gut microbiota and nutrient loading/calorie intake [7, 41]. Since the advent of cooking, ancestral humans gradually adapted to diversified TF and therefore experienced an increased access to energy-rich food. Unexpectedly, recent lifestyle change has negative impacts on the so-called “forgotten organ,” the human gut microbiota [42]. Thus, gut microbiota may in turn act on the adaptations of host physiology and metabolic pathways [43], which eventually contributes to the evolutionary trajectories of host and symbiotic microbiota [44].

In addition, our study also has implications for understanding microbial community differences and variations among populations. Since gut enterotypes firstly proposed based on differential members of gut microbiota associated with dietary characteristics in humans [40], some work, subsequently, indicate strong diet effects on enterotype status [44–47], but lack consensus of enterotypes [48]. In two studies focusing on mice fed a similar diet by Wu et al. [45] and Wang et al. [47], despite the clear evidence for contributions of a long-term dietary history on enterotypes, the opposite effects of *Bacteroides*-dominant enterotypes were observed in a short term. A very recent study has revealed that enterotypes have no capabilities of reflecting resident microbial communities across diverse human populations, nor were they able to effectively distinguish the communities when those reanalyzed by removing their *Bacteroides* and *Prevotella* members [49]. The *Bacteroides*-dominant enterotype dominates when diet rich in animal protein and fat are consumed, whereas the *Prevotella*-dominant enterotype is believed to be prevalent in individuals with high carbohydrate diets [45]. However, in this study, even though supplied with the same food source, mice with TF had dramatically a lower abundance level of *Bacteroides* compared to those with NF. This means that if enterotype-like clusters continue to be driven by a key microbial taxon, our finding would further highlight the gaps in enterotype categorization. Despite the controversies, these results illustrate that gut microbiota are driven not only by host diet but also by the evolution of feeding ecology and life history [50]. Taken together, substantial gut microbial shifts may have occurred during the human evolution via adaptation to dietary transitions in terms of both foodstuffs and their processing methods such as thermal processing, concurrently affecting the mutualistic interactions of the host-gut microbiota. Likewise, whether gaps in the *Prevotella*-dominant enterotype occur in host feeding on plant-based TF is worth exploring further.

## Conclusions

Ecological and evolutionary factors, such as dietary components and lifestyles, have been used to disentangle host-gut microbiota adaptations and interplay, though the effect of thermal processing associated with food preparations on gut microbiota has been unexplored so far. Our data indicate, in addition to host genotype and diet, thermal processing that significantly reduced gut microbial diversity and altered the communities in fish and mice. We found opposite patterns of changes in many predominant microbial compositions between fish and mice, suggesting that specific adaptive trajectory of host-gut microbiota driven by TF in aquatic and terrestrial animals might be thoroughly specialized within host populations from the perspectives of a long-term evolutionary history. These results would be beneficial for elucidating decreases of microbial diversity and changes of community structure. Given the prevalence of thermal processing of food worldwide, the difference in food processing, such as boiled, baked and steamed, and the potentially profound effect of non-food factors on gut microbial community and function, best practice currently would appear to undertake further studies on the mechanisms underlying by which how TF modulates gut microbial communities, more in particular quantify the effect size of different thermal processing techniques, from daily life to lifespan scales, combining with habitual food on gut microbiota.

## Additional files

Additional file 1: This file contains all the supporting information that is associated with the manuscript, including four additional figure captions and legends and four additional tables. The figures are included in separate files and labeled Figures S1–S4. (ZIP 1360 kb)

## Abbreviations

DNA: Deoxyribonucleic acid
F_NG: Fish fed non-thermally processed grass carp
F_NS: Fish fed non-thermally processed stone moroko
F_TG: Fish fed thermally processed grass carp
F_TS: Fish fed thermally processed stone moroko
LDA: Linear discriminant analysis
LEfSe: Linear discriminant analysis effect size
M_BA: Mice before experimental food intervention
M_NG: Mice fed non-thermally processed grass carp
M_TG: Mice fed thermally processed grass carp
NF: Non-thermally processed food
NG: Non-thermally processed grass carp
NS: Non-thermally processed stone moroko
PCA: Principal component analysis
PCoA: Principal coordinate analysis
PERMANOVA: Permutational multivariate analysis of variance
rRNA: Ribosomal ribonucleic acid
TF: Thermally processed food
TG: Non-thermally processed grass carp
TS: Non-thermally processed stone moroko
