# Supplementary material for: Thermal processing of food reduces gut microbiota diversity of the host and triggers adaptation of the microbiota: evidence from two vertebrates
Source: Microbiome. 2018 May 31;6:99. doi: 10.1186/s40168-018-0471-y (PMC5984331; doi:10.1186/s40168-018-0471-y)
Supplement: Supplementary file 1 — This file contains all the supporting information that is associated with the manuscript, including four additional figure captions and legends and four additional tables. The figures are included in separate files and labeled Figures S1–S4. (ZIP 1360 kb) [file 40168_2018_471_MOESM1_ESM.zip › Additonal figure legends and tables.docx]

**Supporting information**

**Thermal processing of food reduces gut microbiota diversity of the host and triggers adaptation of the microbiota: evidence from two vertebrates**

**Zhimin Zhang^1,2^ and Dapeng Li^1,2,*^**

^1^Department of Fishery Resources and Environment, College of Fisheries, Huazhong Agricultural University, Wuhan, P.R. China

^2^Hubei Provincial Engineering Laboratory for Pond Aquaculture, Wuhan, P.R. China

**^*^**Corresponding author: Dr. Dapeng Li

E-mail address: [ldp@mail.hzau.edu.cn](mailto:ldp@mail.hzau.edu.cn)

First author: Dr. Zhimin Zhang

E-mail address: [zhzhmin@webmail.hzau.edu.cn](mailto:zhzhmin@webmail.hzau.edu.cn)

**Additional tables**

**Table S1.** Body weight of fish and mice at the end of the experiments.

| Tank ID | Fish ID | Body weight (g) | Cage ID | Mice ID | Body weight (g) |
| --- | --- | --- | --- | --- | --- |
| 1 | F-TG 1 | 123.7 | 1 | M-TG 1 | 25.8 |
|  | F-TG 2 | 93.9 |  | M-TG 2 | 25.1 |
| 2 | F-TG 3 | 120.5 | 2 | M-TG 3 | 23.2 |
|  | F-TG 4 | 119.0 |  | M-TG 4 | 24.9 |
| 3 | F-NG 1 | 153.4 | 3 | M-NG 1 | 22.3 |
|  | F-NG 2 | 140.9 |  | M-NG 2 | 23.0 |
| 4 | F-NG 3 | 151.3 | 4 | M-NG 3 | 21.5 |
|  | F-NG 4 | 142.6 |  | M-NG 4 | 20.1 |
| 5 | F-TS 1 | 124.6 |  |  |  |
|  | F-TS 2 | 111.0 |  |  |  |
| 6 | F-TS 3 | 127.1 |  |  |  |
|  | F-TS 4 | 108.6 |  |  |  |
| 7 | F-NS 1 | 142.2 |  |  |  |
|  | F-NS 2 | 159.3 |  |  |  |
| 8 | F-NS 3 | 152.5 |  |  |  |
|  | F-NS 4 | 144.3 |  |  |  |

**Table S2.** Proximate composition of non-thermally and thermally processed grass carp fillets (NG and TG) and stone moroko (NS and TS).

| Proximate  composition | Grass carp fillets | | Stone moroko | |  |
| --- | --- | --- | --- | --- | --- |
|  | NG | TG^#^ | NS | TS^#^ | *p*^$^ |
| Moisture | 80.24 ± 0.30 | 72.55 ± 0.89^***^ | 79.92 ± 0.63 | 75.45 ± 0.97^***^ |  |
| Fat | 2.20 ± 0.18 | 2.25 ± 0.6 | 2.28 ± 0.44 | 2.27 ± 0.33 |  |
| Protein | 15.99 ± 0.22 | 14.89 ± 0.26^**^ | 13.41 ± 0.43 | 12.6 ± 0.82 | ^***^ |
| Ash | 1.29 ± 0.10 | 1.23 ± 0.10 | 3.11 ± 0.23 | 3.17 ± 0.20 | ^***^ |

Values are means (n=4, individual samples per treatment) with standard deviations (SD). ^#^Moisture is based on the thermally-processed food, whereas fat, protein and ash are presented as the percentages of fresh food (non-thermally-processed food, wet weight). NG and TG represent non-thermally and thermally processed grass carp fillets, respectively. NS and TS represent non-thermally and thermally processed stone moroko, respectively. ^$^The comparisons between grass carp fillets and stone moroko. The asterisk indicates significant differences between pair-wise treatment groups or between grass carp fillets and stone moroko, ^*^ *p* < 0.05, ^**^ *p* < 0.01 and ^***^ *p* < 0.001. (Data cited from Zhang et al. 2018)

**Table S3.** Two-way PERMANOVA based on unweighted UniFrac distance testing whether gut microbial communities have differences between mice and catfish fed non-thermally and thermally processed grass carp fillets.

| Source | d.f. | SS | MS | Pseudo-*F* | *p* |
| --- | --- | --- | --- | --- | --- |
| Host | 0.045061 | 1 | 0.045061 | 12.704 | 0.0001 |
| Treatment | 0.009413 | 1 | 0.009413 | 2.6537 | 0.0217 |
| Interaction | 0.020877 | 1 | 0.020877 | 5.8857 | 0.0002 |
| Residual | 0.042564 | 12 | 0.003547 |  |  |
| Total | 0.11791 | 15 |  |  |  |

**Table S4.** Two-way PERMANOVA based on unweighted UniFrac distance testing whether gut microbial communities have differences in catfish fed non-thermally and thermally processed grass carp fillets and stone moroko.

| Source | d.f. | SS | MS | Pseudo-*F* | *p* |
| --- | --- | --- | --- | --- | --- |
| Treatment | 0.045401 | 1 | 0.045401 | 10.37 | 0.0001 |
| Food | 0.012972 | 1 | 0.012972 | 2.963 | 0.0173 |
| Interaction | 0.011196 | 1 | 0.011196 | 2.5572 | 0.0346 |
| Residual | 0.052537 | 12 | 0.004378 |  |  |
| Total | 0.12211 | 15 |  |  |  |

**Additional figure legends**

**Fig. S1** Principal Component Analysis based on profiles of a) fatty acids and b) amino acids in four groups of experimental foods. NG and TG represent non-thermally and thermally processed grass carp fillets, respectively. NS and TS represent non-thermally and thermally processed stone moroko, respectively. (Data cited from Zhang et al. 2018)

**Fig. S2** Microbial composition and community structure of the experimental food. (A) The relative abundance of microbial composition at the phylum level; (B) The relative abundance of microbial composition at the genus level; (C) Principal Coordinate Analysis based on weighted UniFrac distance for food microbiota at the OTU level.

**Fig. S3** Microbial composition and community structure in mice before and after food intervention. (A) Principal Coordinate Analysis based on weighted UniFrac distance for gut microibota at the OTU level; (B) The weighted UniFrac distance within groups; (C) The relative abundance of microbial composition at the phylum level; (D) The relative abundance of microbial composition at the genus level.

**Fig. S4** Effects of thermal processing of food on *Firmicutes*-*Bacteroidetes* ratios in the gut of the catfish and mice.

**Reference**

Zhang Z, Xu W, Tang R, Li L, Refaey MM, Li D. Thermally processed diet greatly affects profiles of amino acids rather than fatty acids in the muscle of carnivorous Silurus meridionalis. Food Chemistry, 2018, 256:244-251.
