# Supplementary figures and images for: Thermal processing of food reduces gut microbiota diversity of the host and triggers adaptation of the microbiota: evidence from two vertebrates
Source: Microbiome. 2018 May 31;6:99. doi: 10.1186/s40168-018-0471-y (PMC5984331; doi:10.1186/s40168-018-0471-y)

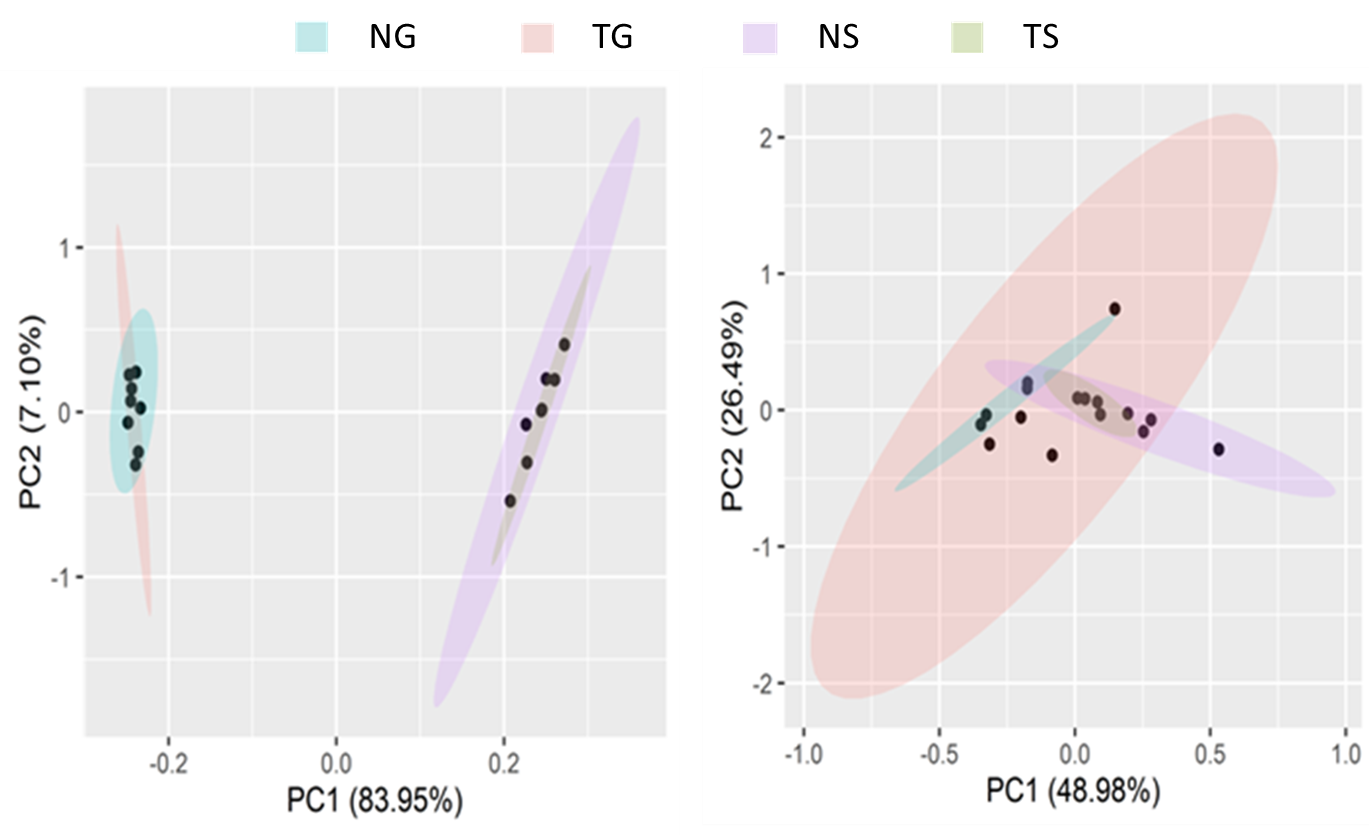

Supplement: Supplementary file 1 — This file contains all the supporting information that is associated with the manuscript, including four additional figure captions and legends and four additional tables. The figures are included in separate files and labeled Figures S1–S4. (ZIP 1360 kb) [file 40168_2018_471_MOESM1_ESM.zip › Figure S1.tif]
